# Understanding the Effects of Antecedents on Continuance Intention to Gather Food Safety Information on Websites

**DOI:** 10.3389/fpsyg.2020.579322

**Published:** 2020-12-11

**Authors:** Hsinyeh Tsai, Yu-Ping Lee, Athapol Ruangkanjanases

**Affiliations:** ^1^Department of Business Administration, Shu-Te University, Kaohsiung, Taiwan; ^2^Chulalongkorn Business School, Chulalongkorn University, Bangkok, Thailand

**Keywords:** food safety, social media, continuance intention, expectation-confirmation theory, technology acceptance model, technology readiness

## Abstract

Virtual community websites are one of the applications that provide a platform for people with common interests to extend their social relations in social media. With the proliferation of food safety incidents in recent years, social media has often been a major channel for public engagement in risk communication because of its social networking and immediate interaction. To understand the users’ needs and satisfaction, this study proposed a model to develop and evaluate the antecedents of continuance intention toward food safety information from social media. Based on the questionnaire collected from 289 Facebook users, this study assessed the integrated model of the expectation-confirmation theory and technology acceptance model with technology readiness as moderator. The results showed that the perceived ease-of-use, usefulness, and confirmation indirectly affected social media continuance usage intention through satisfaction; perceived ease-of-use, usefulness, and satisfaction were the direct determinants that affected the users’ social media continuance intention. Furthermore, positive technology readiness had significant effects on the relationship between the perceived ease-of-use, usefulness, confirmation, satisfaction, and continuance intention toward food safety information. This study contributes some important suggestions and managerial implications for food safety promotion providers, practitioners, and academics in the food industry, and social media environment.

## Introduction

Beyond traditional media, such as television, newspapers, and magazines, social media has gained unprecedented popularity in food safety issues and became another major source of food safety information for people today (Rutsaert et al., 2014). Food safety is the focus of public health and people’s livelihood, and the succession of food safety incidents in the recent years has caused consumers to distrust food safety, causing huge economic losses. Thus, the inexorable rise of social media to challenge, and even replace, the functions, and roles of traditional media can perhaps be attributed to several obvious innate characteristics. On the one hand, it absorbs users as much as possible and provides an immediate, participatory, continuous, and collaborative mode of interaction for each user (Ding, 2009). On the other hand, it facilitates rapid circulation and exchange of user-generated content over time (Macias et al., 2009).

Today’s so-called media consists of blogs, citizen journalists, Facebook and Twitter users, and traditional media; thus, anyone can participate in the information production process. Such a change has drastically altered the tradition of issue setting, and issues that have received media attention in the past will influence whether the public gives the issue the same attention. However, with the advent of Web 2.0 and social media, the effectiveness of traditional media in influencing public opinion has been greatly affected. Since social media platforms have a much lower barrier to participation than many traditional media platforms, the average person can engage in public dialogue through social platforms. Hence, traditional media must adopt a more flexible business model that adapts to the changing media ecosystem and leverages some of the qualities of social media to gain market and reader attention. Therefore, this study aimed to focus on Taiwanese consumers’ perceptions, attitudes, demands, consumption behaviors, and their correlations with organic food, and identify the factors that influence consumers’ purchase behavior of organic food.

The so-called “organic food” without chemical residues has gradually became another trend in recent years. Due to the emergence of organic food and the demand for health and safety concepts, consumer acceptance of organic food is gradually increasing. Whether the quality and price of organic food in the market meet the consumers’ expectations or needs are a matter of concern. Previous research on Facebook has focused only on user or website characteristics (Acquisti and Gross, 2006; Dwyer et al., 2007; Mazer et al., 2007; Ross et al., 2009). These characteristics included user social experience, motivation, personality, privacy, and self-disclosure. Recently, some studies began focusing on the users’ continuance behavior to use Facebook (Lee et al., 2012). Since social media is a type of information system, the success of the system depends on the user’s continuance use behavior and thus, understanding the users’ needs becomes the most urgent issue. This study composed an expectation-confirmation model (ECM) as a part of the research model. ECM theorized that consumers had expectations before purchasing a product.

Bhattacherjee (2001) believed that the IT users’ continuance use intention was like the consumers’ repurchase decision-making behavior. Before using social media, users had expectations about their way of interaction, interests sharing, and establishing personal networks. After using social media, users would compare their actual feelings with previous expectations. If their actual feelings were better than their expectations, they would have higher satisfaction and continue to use it. To predict and explain the users’ continuance to use the system more effectively, Bhattacherjee (2001) modified the expectation confirmation theory and proposed a post-acceptance model of IS continuance.

Technology readiness measures people’s tendency to accept or use new technologies, especially the acceptance of networks or computers. Parasuraman (2000) summarized people’s positive and negative feelings toward technology, which were applicable to different groups (consumers, companies, and employees). Liljander et al. (2006) confirmed in his study that technology readiness was crucial for users’ acceptance of innovative technology.

Some previous research on social media, such as Facebook, focused only on the users’ personal factors or site characteristics. Important factors, such as technology readiness, were ignored. Technology readiness affects not only the users’ acceptance of a system but also their acceptance of new products or services (e.g., Yen, 2005; Lin and Hsieh, 2007; Chen et al., 2009, 2013; Chen, 2012; Jin, 2013). However, the technology acceptance model (TAM) only considers the perception of usefulness and ease of use, without considering that personal characteristics will also affect one’s willingness to use technology products, that is, whether the user will accept or use the new technology, influenced by his or her accumulated experience and knowledge personality tendency, which ultimately depends on whether the individual has the sufficient technology readiness. Therefore, this study adopted the ECM proposed by Bhattacherjee (2001), TAM proposed by Davis et al. (1989), and technology readiness by Parasuraman (2000) as an antecedent to explain and predict the influence of technology readiness on users’ continuance intention to use social media for food safety.

## Literature Review

### Expectation-Confirmation Model

In the field of studying consumer behavior, the expectation confirmation theory has been widely adopted to assess consumers’ satisfaction and repurchase behavior (Oliver, 1980, 1993; Tse and Wilton, 1988; Anderson and Sullivan, 1993; Patterson et al., 1997; Dabholkar et al., 2000). The main concept of expectation confirmation theory was that consumers would confirm their pre-purchase expectation with post-purchase perceived performance to determine their level of satisfaction and then influence their repurchase intention.

Bhattacherjee (2001) suggested that the users’ continuance intention of information systems was like the consumers’ repurchase decisions. However, the use of information systems was not the same as purchasing behavior in practice. Thus, Bhattacherjee (2001) modified the expectation disconfirmation theory and proposed ECM. The main idea of ECM is that the information system continuance is affected by satisfaction, perceived usefulness, and expectation-confirmation. Lee (2010) applied ECM to explain and predict the e-learning users’ continuance intention. Kim (2010) used ECM to forecast mobile data service continuance. Both studies combined ECM with TAM. The results showed that the extension of ECM had a good explanatory power for the continued use of IT. Previous studies have proven that the users’ perceived ease-of-use and confirmation affects their level of satisfaction (Hsu et al., 2006; Roca et al., 2006; Limayem and Cheung, 2008; Yu, 2010; Chen et al., 2018; Hsiao, 2018; Kim et al., 2019; Dai et al., 2020; Gupta et al., 2020; Park, 2020; Tam et al., 2020). Furthermore, satisfaction influences the continuance intention to use (Hsu et al., 2006; Roca et al., 2006; Wu et al., 2007; Chen and Zhou, 2008; Chen et al., 2009; Jin et al., 2009; Yu, 2010).

To understand the user behavior of food safety on social media, this study used Facebook fan pages and groups as the main body of research and applied ECM as a theoretical basis to construct the behavior pattern of the food safety community to understand the relevant factors that affect the consumers’ intention to continue using it.

### Technology Acceptance Model (TAM)

TAM (Davis et al., 1989) used users’ attitudes as a major factor for predicting their behavior intention, whether to accept a new information technology. Additionally, the perceived usefulness and perceived ease-of-use were included in this model as determinant factors of attitude. However, TAM investigated human behavior intention only at the cognitive level, ignoring the influences of external factors (Davis, 1989). Davis (1989) suggested that researchers extend TAM with external variables based on different theories to discuss different technology acceptance.

Since TAM was used to predict users’ technology acceptance in the working environment, Lin et al. (2007) were concerned about the adoption of TAM in the marketing setting. Rauniar et al. (2014) presented the results for the importance of perceived ease-of-use and usefulness with additional some determinants variables to predict user engagement on social media platforms. Previous studies also provided evidence about the two key influencers (i.e., perceived usefulness and ease-of-use) to understand the impacts of antecedents on behavioral intention toward social media (Ayeh, 2015; Chang et al., 2015; Lin and Kim, 2016; Luo et al., 2019). Therefore, our study revised TAM and added external variables to enhance the interpretive abilities to gather food safety information toward social media.

### Technology Readiness

Technology readiness is the tendency of people to accept and use new technologies at home or work to achieve their goals (Parasuraman, 2000). Technology readiness is an individual personality trait, and this concept can be seen as an overall psychological state of an individual’s tendency to use new technologies, as determined by the psychological aspects of positive drivers (positive readiness) and inhibitors (negative readiness). Positive drivers encompass optimism and innovativeness while negative inhibitors encompass discomfort and insecurity. Tsikriktsis (2004), in an extended study of technology readiness, also noted that consumers with different levels of technology readiness have different intentions for current and future use of information services.

Later, Parasuraman and Colby (2015) proposed an updated and streamlined TRI 2.0, which is simplified into 16 topics that can be tiered to manage customers with different technology readiness. Since technology readiness is a measure of an individual’s psychological inclination and personal characteristics toward new technologies or services, this study considers that the level of technology readiness of an individual will be an important antecedent for consumers to use social media to explore food safety issues.

In the relevant studies regarding technology readiness and other theoretical model, Lin et al. (2007) proposed the Theory of Technology Readiness and Acceptance Model (TRAM), to increase the explanatory power of TAM on the customers’ acceptance of innovative technology. Technology Readiness refers to the people’s tendency to accept and use new technologies for accomplishing goals at home or work. Thus, technology readiness could be viewed as an individual’s psychological tendencies to use new technologies. Prior studies have confirmed that technology readiness could combine other theoretical model to explain the adoption of information systems and technologies (Lin and Hsieh, 2006, 2007; Lin et al., 2007; Walczuch et al., 2007; Chen et al., 2009, 2013; Lin and Chang, 2011; Jin, 2013). It, therefore, seemed reasonable to consider the elements of technology readiness to investigate the adoption of social media to update user’s food safety information.

## Research Methods

### Hypotheses Development and Research Model

According to the definition of Parasuraman (2000), technology readiness measures a user’s readiness to adopt new technology/system based on four personality traits: optimism, innovativeness, discomfort, and insecurity. Of the four traits, optimism and innovativeness are positive drivers of technology readiness (i.e., positive technology readiness) while discomfort and insecurity are combined as the inhibitors (i.e., negative technology readiness). Jin (2013) validated that the perception of ease-of-use and usefulness were influenced by the users’ positive technology readiness in social media usage. Chen et al. (2009) empirically showed that optimism and innovativeness of technology readiness was a strong predictor on satisfaction in self-service technologies. Prior research also examined an integrated model of technology readiness into TAM and found that technology readiness provided the positive linkage with the perceptions of usefulness and ease-of-use (Lin et al., 2007). Furthermore, the positive correlations from technology readiness to perceived usefulness, satisfaction, and continuance intention also were confirmed in mobile service (Chen et al., 2013). Based on the above literature, we proposed the following research hypotheses:

H1: Positive technology readiness positively influences the perceived ease-of-use.

H2: Positive technology readiness positively influences perceived usefulness.

H3: Positive technology readiness positively influences satisfaction.

H4: Positive technology readiness positively influences continuance intention.

H5: Negative technology readiness positively influences the perceived ease-of-use.

H6: Negative technology readiness positively influences perceived usefulness.

H7: Negative technology readiness positively influences satisfaction.

H8: Negative technology readiness positively influences continuance intention.

Davis (1989) illustrated the perceived ease-of-use to the extent to which a user believes that using a particular technology/system would be effortless. Previous studies regarding information technology adoption have found that the higher the perceived ease-of-use, the higher of their perceptions of usefulness (Chen et al., 2009, 2018; Liu and Li, 2011; Mehrad and Mohammadi, 2017; Wu and Chen, 2017; Verma and Sinha, 2018). Information technology acceptance research has consistently indicated that the perceived ease-of-use is the critical influencer of users’ satisfaction (Liao et al., 2007; Bienstock et al., 2008; Chen et al., 2009; Recker, 2010; Shin et al., 2011; Joo et al., 2018). Therefore, investigating the perceived ease-of-use and its effect on perceived usefulness and satisfaction seems necessary.

H9: Perceived ease-of-use positively influences perceived usefulness.

H10: Perceived ease-of-use positively influences satisfaction.

Bhattacherjee (2001) proposed the ECM to evaluate the three determinants of information technology continuance intention as follows: the extent of confirmation of expectations, perceived usefulness, and level of individual’s satisfaction. Existing empirical evidence on social media has shown that users’ continuance intention is mainly determined by their satisfaction with prior social media usage (Chen, 2012). In social media contexts, Oghuma et al. (2016) empirically indicated the fact that satisfaction was predicted by the degree of confirmation and perceived usefulness of mobile instant messaging services. Similarly, other studies have consistently illustrated the positive linkage among confirmation, perceived usefulness, and satisfaction (Thong et al., 2006; Chen et al., 2013; Dai et al., 2020; Tam et al., 2020). Therefore, this study proposed the following hypotheses:

H11: Satisfaction positively influences continuance intention.

H12: Confirmation positively influences perceived satisfaction.

H13: Confirmation positively influences perceived usefulness.

H14: Perceived usefulness positively influences satisfaction.

H15: Perceived usefulness positively influences continuance intention.

**FIGURE 1:**
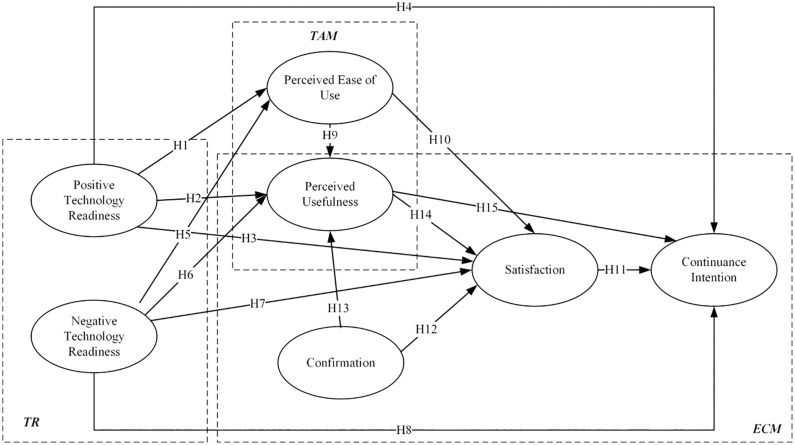
Research model.

The research model of this study is shown in Figure 1 and research hypotheses are presented in Table 1.

**TABLE 1:** Research hypotheses.

Hypothesis	Hypothesis description
Hypothesis 1	Positive technology readiness positively influences perceived ease-of-use
Hypothesis 2	Positive technology readiness positively influences perceived usefulness
Hypothesis 3	Positive technology readiness positively influences satisfaction
Hypothesis 4	Positive technology readiness positively influences continuance intention
Hypothesis 5	Negative technology readiness negatively influences perceived ease-of-use
Hypothesis 6	Negative technology readiness negatively influences perceived usefulness
Hypothesis 7	Negative technology readiness negatively influences satisfaction
Hypothesis 8	Negative technology readiness negatively influences continuance intention
Hypothesis 9	Perceived ease-of-use positively influences perceived usefulness
Hypothesis 10	Perceived ease-of-use positively influences satisfaction
Hypothesis 11	Satisfaction positively influences continuance intention
Hypothesis 12	Confirmation positively influences perceived satisfaction
Hypothesis 13	Confirmation positively influences perceived usefulness
Hypothesis 14	Perceived usefulness positively influences satisfaction
Hypothesis 15	Perceived usefulness positively influences continuance intention

### Questionnaire Design

The research questionnaire included three segments. The latent variables in this study used a seven-point Likert scale drawn and modified from existing qualified and relevant studies, where 1 meant strongly disagree and 7 meant strongly agree. The measurement scales in the first part were to determine users’ perceptions when using Facebook about the issues of food safety information, including perceived usefulness, perceived ease-of-use, and confirmation of expectations. Perceived usefulness and ease-of-use, with four items, respectively, were measured adopting four items modified from Abdullah et al. (2016) and Joo et al. (2018). The second part was to understand users’ satisfaction and continuance intention to use Facebook for gather food safety information. Satisfaction and continuance intention were based on Bhattacherjee (2001) and Chen et al. (2013) with four items individually. The third part was to test the users’ technology readiness including 18 items from Yen (2005) and Chen et al. (2013). Technology readiness included the positive drivers (optimism and innovativeness) and negative inhibitors (discomfort and insecurity). Furthermore, two university professors and five master students who are well acquainted with social media behavior got together as a focus group to assist to assess the appropriateness of the questionnaire items in this study.

### Data Collection

Data was collected online from users who had previously used Facebook in Taiwan. The selected sample in this study is the current potential consumers owned by four famous Facebook Fan Pages and groups about promoting food safety information in Taiwan (i.e., @Learneating^1^; @foodnext.net^2^; @FoodQA^3^; @wayneagrifoodlife^4^). In the four representative Facebook Fan Pages, each fan page has more than 10,000 potential users and consumers in the groups. Recruiting these experienced and potential consumers from Facebook Fan Pages about promoting food safety information would facilitate external validity. For all 318 questionnaires received, 289 were valid. Among them, 44.80% were male and 55.20% were female. Furthermore, 22.93% were aged under 20 years, 61.31% were 21–30 years, 12.35% were 31–40 years, and 3.41% were above the age of 41 years.

## Results

### Measurement Model Analysis

This study adopted the partial least squares (PLS) method and applied the SmartPLS 3.3.2 software developed by Ringle et al. (2015). PLS is a structural equation model (SEM) analysis technique based on regression analysis. PLS is highly practical and superior to general linear relational model analysis techniques, which can handle both reflective and formative model structures. The requirements for variables to conform to normal distribution, randomness, and sample size are less stringent. In addition, PLS can overcome multicollinearity problems, effectively handle interference, and missing values, and has good predictive and explanatory capabilities. Since the sample size of this study is small, the analysis using PLS is not limited by sample size and variable allocation type and has good prediction and interpretation ability. Bootstrap resampling was conducted 5,000 times (Chin, 1998) to ensure the stability of the estimation of each variable.

Petter et al. (2007) argued that the analytical approach to PLS was largely based on the component-based model, while traditional SEM was based on the covariance-based model as a broad alternative to the covariance-based model, and the component-based model was a good alternative to the covariance-based model. The measurement model of the research tool and research variables can be examined simultaneously. The analysis and estimation of PLS were divided into two stages: the first stage was to perform reliability and validity analysis on the measured model and the second stage was to perform path coefficient verification and model prediction capability estimation on the structural model. Such an estimation procedure was used to test the reliability and validity of the variables, that is, to confirm the appropriateness of the explanations of the variables, and then test, and specify the relationships among the variables to validate the assumptions of the research framework (Hulland, 1999).

Convergent validity refers to the measures of constructs that are expected to correlate to a certain degree. This study used composite reliability (CR) and average variance extracted (AVE) to establish convergent validity (Hair et al., 1998). CR assessed the internal consistency of a measure. A higher CR value represented better internal consistency. Fornell and Larcker (1981) suggested that the CR value should be 0.6 or better. AVE refers to the explanatory power of the latent variable to other measured variables. If the corresponding AVE values of the constructs were 0.5 or above, convergent validity existed (Fornell and Larcker, 1981). All AVE values are higher than 0.5, and CR values are higher than 0.7. All constructs in this study had good convergent validity (as shown in Table 2).

**TABLE 2:** Reliability and average variance extracted.

Variable	Composite reliability	Cronbach’s Alpha	Average variance extracted
POS_TR	0.93	0.92	0.56
NEG_TR	0.87	0.83	0.54
PEOU	0.91	0.87	0.73
CON	0.89	0.82	0.74
PU	0.92	0.88	0.73
SAT	0.93	0.89	0.76
CINT	0.92	0.89	0.74

**POS_TR, Positive Technology Readiness; NEG_TR, Negative Technology Readiness; PEOU, Perceived Ease-of-Use; CON, Confirmation; PU, Perceived Usefulness; SAT, Satisfaction; CINT, Continuance Intention.**

Discriminant validity examined the degree of differences among the constructs. Fornell and Larcker (1981) pointed out that the AVE of each construct should be greater than the squared correlation coefficient value of paired constructs. In other words, the AVE’s square root should be greater than the correlation value among constructs. As shown in Table 3, the empirical results indicated each square root degree of AVE in one of the specific constructs are higher than all the correlation coefficients between the construct and other constructs. Besides, in Table 4, all the coefficients of the heterotrait-monotrait ratio of correlations (HTMT) were below the threshold of 0.85 (Henseler et al., 2016). These results provided sufficient evidence of discriminant validity for the proposed model in this study.

**TABLE 3:** Correlation matrix.

	POS_TR	NEG_TR	PEOU	CON	PU	SAT	CI
POS_TR	***0.75***						
NEG_TR	–0.21	***0.73***					
PEOU	0.37	–0.18	***0.85***				
CON	0.45	0.63	–0.08	***0.86***			
PU	0.51	–0.16	0.62	0.64	***0.86***		
SAT	0.50	–0.09	0.64	0.73	0.67	***0.87***	
CI	0.44	–0.14	0.72	0.69	0.69	0.70	***0.86***

**POS_TR, Positive Technology Readiness; NEG_TR, Negative Technology Readiness; PEOU, Perceived Ease-of-Use; CON, Confirmation; PU, Perceived Usefulness; SAT, Satisfaction; CINT, Continuance Intention. The non-diagonal value represents the correlation coefficient of each component. The value of the diagonal represents the square root of the Average Variance Extraction (AVE) value of the component.**

**TABLE 4:** HTMT for discriminant validity.

	POS_TR	NEG_TR	PEOU	CON	PU	SAT	CI
POS_TR							
NEG_TR	0.23						
PEOU	0.37	0.19					
CON	0.48	0.13	0.74				
PU	0.49	0.17	0.71	0.73			
SAT	0.49	0.10	0.72	0.84	0.75		
CI	0.42	0.14	0.82	0.80	0.79	0.78	

**POS_TR, Positive Technology Readiness; NEG_TR, Negative Technology Readiness; PEOU, Perceived Ease-of-Use; CON, Confirmation; PU, Perceived Usefulness; SAT, Satisfaction; CINT, Continuance Intention.**

This study used a self-reported questionnaire to collect the Facebook users’ responses in Taiwan. Since one questionnaire to sample the whole population might have common method bias (CMB), Harman’s one-factor test was conducted to examine the data. The basic assumption of Harmon’s single factor test was that when one single factor could explain most covariance among all variables, CMB existed. In this study, all variables were subjected to principal component analysis (PCA) for factor analysis. Seven factors with eigenvalues greater than 1 were extracted from 35 questions. The cumulative explained variance for these six factors was 69.53%, and the first factor could explain 18.49% variance, less than 50%. Thus, CMB did not exist significantly in this study.

### Structural Model

In this study, path analysis was used to examine the substantive relationships among the variables after confirming that the measurement components met the requirements of acceptable reliability and validity. For overall path analysis, PLS was used in this study to examine the relationships between variables, bootstrapping was used to estimate the path coefficients, and re-sampling of the data was used to estimate the value, which was a better approximation than the commonly used limit. Therefore, this study adopted this method to investigate the relationship between variables. The structural model and path analysis results are shown in Table 5. The results of the PLS analysis showed that the components of the proposed framework in this study affected the continuance intention including perceived usefulness (β = 0.39, *p* < 0.001) and satisfaction (β = 0.42, *p* < 0.001). However, the effect of POS_TR (β = 0.04, *P* = 0.45) and NEG_TR (β = 0.00, *P* = 0.93) on the continuance intention was not significant. In the antecedents on satisfaction of each component, the effect of confirmation (β = 0.41, *p* < 0.001), POS_TR (β = 0.14, *p* < 0.01), perceived usefulness (β = 0.22, *p* < 0.001), and perceived ease of use (β = 0.20, *p* < 0.001) on satisfaction was significant, but the effect of NEG_TR (β = 0.04, *p* > 0.05) on satisfaction was not significant. Furthermore, POS_TR had the direct effects on perceived usefulness (β = 0.34, *p* < 0.001) and perceived ease-of-use (β = 0.23, *p* < 0.001) were significant. However, the effect of NEG_TR on perceived usefulness (β = −0.11, *p* > 0.05) and perceived ease-of-use (β = −0.03, *p* > 0.05) were insignificant.

**TABLE 5:** Research hypotheses testing.

Hypothesis	Path	Standardized path coef.	Standard deviation	T statistics
H1	POS_TR -> PEOU	0.34***	0.06	5.70
H2	POS_TR -> PU	0.23***	0.05	4.32
H3	POS_TR -> SAT	0.14**	0.05	2.88
H4	POS_TR -> CI	0.03	0.05	0.49
H5	NEG_TR -> PEOU	−0.11	0.06	1.75
H6	NEG_TR -> PU	−0.03	0.05	0.62
H7	NEG_TR -> SAT	0.04	0.04	0.90
H8	NEG_TR -> CI	−0.03	0.04	0.70
H9	PEOU -> PU	0.34***	0.06	5.21
H10	PEOU -> SAT	0.20***	0.06	3.54
H11	SAT -> CI	0.42***	0.06	7.33
H12	CON -> SAT	0.41***	0.06	6.91
H13	CON -> PU	0.32***	0.07	4.77
H14	PU -> SAT	0.22***	0.06	3.91
H15	PU -> CI	0.39***	0.06	6.84

POS_TR, Positive Technology Readiness; NEG_TR, Negative Technology Readiness; PEOU, Perceived Ease-of-Use; CON, Confirmation; PU, Perceived Usefulness; SAT, Satisfaction; CINT, Continuance Intention. *p < 0.05; **p < 0.01; ***p < 0.001.

## Conclusion

### Theoretical Contribution

Social media is the messaging system of today’s society at the outbreak of a major food safety crisis and is a forum where public opinion can be expressed in real time. This study analyzed factors (perceived usefulness, perceived ease of use, confirmation, and satisfaction) that affect the continuance intention of social media users. The results indicated that the proposed research model not only passed the examination of convergent validity, discriminant validity, and model fits, but also had a higher explanatory power than the post-acceptance model of IS continuance proposed by Bhattacherjee (2001). That is, by adding the factor of perceived ease of use, the research model had better prediction of perceived usefulness, satisfaction, and continuance intention of social media users. Perceived ease of use had a positive impact on the users’ perceived usefulness and continuance intention. This finding is consistent with many extended TAM research (Dabholkar, 1996; Hair et al., 1998; Dabholkar and Bagozzi, 2002; Kleijnen et al., 2004; Lin and Hsieh, 2007; Walczuch et al., 2007; Ross et al., 2009). The ease-of-use user interface of a social media website allows users to accomplish their purposes easily that also increased their perceived usefulness of the website, affecting their continuance intention to use it.

### Practical Contribution

Due to the increased accessibility of the Internet in households and the high adoption of Web 2.0 technology in websites, social media has become widespread. Social media is a platform to build social networks or social relations among people who share common interests, activities, backgrounds, or real-life connections. Most social media are run on websites offering different functions for user interactions. The functions include chatting, email, uploading photos, sharing links and videos, blogging, and discussion groups. Through these interactions, social media can connect people or groups with the same interests or activities and then generate a vast personal network. The nature of social media makes it crucial to play a key role in the coverage of key food safety issues, especially in times of major crisis. While traditional media are often criticized for their limited and homogeneous sources, social media is increasingly seen as a platform for conveying opinions and multiple perspectives on social issues (Guo and Saxton, 2014). While social media does operate for commercial gain, it rarely intervenes in the output of content, as it can be difficult for social media to impose its position on its users and further manipulate the winds of public opinion. Moreover, in terms of the operating model, the benefits of social media are most likely to be in the number of users, not in the sale of homemade content. Compared to traditional media, social media seems closer to being a public discourse arena for social crises. Based on the above findings, the researchers made the following suggestions. First, social media providers should take the users’ needs into consideration when developing new systems. These needs were enhanced system usefulness/functionality, easier-of-use operation interface, and expectation of the users. Although not everyone has the same requirements for social media, not considering any expectations could result in a lower user satisfaction and decrease the possibility of continuance intention to use social media.

Second, social media providers should provide the functions needed by the target users based on their previous experience of using social networking sites (SNS). When developing a new system, social media providers could have volunteers, especially volunteers with low technology readiness, to test drive the system. This would help providers understand the difficulties faced by users with low technology readiness and adjust the system in terms of user interface or system functionality. Therefore, low technology readiness users would find social media easy to access and love using it. In addition, understanding user expectation of social media is also important because system developers cannot bridge the expectation gap without knowing it. By developing the system functionality that users need, their perceived usefulness would increase, resulting in a positive influence on their satisfaction and continuance intention.

Third, this study suggested that perceived usefulness (functionality) and perceived ease of use (brief user interface) influenced both satisfaction and continuance intention of Facebook users. The survey questions regarding perceived usefulness in this study were Facebook functions related to friend interaction and information sharing. Some of the Facebook users complained about the difficulties in searching for friends’ status updates and blogs. Therefore, the researcher suggest that social media providers listen to the needs of the user and upgrade the functionality of the website. When the users’ expectations were met, both their satisfaction and continuance intention could be improved.

### Future Research Directions and Limitations

This study had a few limitations that are as follows. First, compared with traditional and public websites, social media is related earlier to collect users and citizens to discuss the relevant issues about food safety. Thus, the research model in this study could only be applied to free-of-charge social media websites, but not paid or official services. Second, this study discovered the effects of users’ personal characteristics and feelings on their continuance intention to use Facebook. Factors, such as social identification or innovative marketing, that might impact users’ continuance intention could be discussed in future research. The moderating effect of technology readiness was discussed in this study. It is suggested that the other moderating variables can be investigated in a follow-up study. Third, the research objects were drawn from Facebook users in Taiwan; hence, the results were applied in Taiwan only. Because of cultural differences, further research is needed to determine whether the findings in this study are applicable to other countries.

## Data Availability Statement

The raw data supporting the conclusions of this article will be made available by the authors, without undue reservation, to any qualified researcher.

## Ethics Statement

Ethical review and approval was not required for this study on human participants in accordance with the local legislation and institutional requirements. Written informed consent from the patients/participants was not required to participate in this study in accordance with the national legislation and the institutional requirements.

## Author Contributions

HT and Y-PL contributed to research design, empirical analysis, and manuscript writing. AR developed the original idea for the study, proofread, and edited the manuscript. HT, Y-PL, and AR conducted the methodology, data collection, data analysis, and research design. All authors read and approved the final manuscript.

## Conflict of Interest

The authors declare that the research was conducted in the absence of any commercial or financial relationships that could be construed as a potential conflict of interest.

## Footnotes

1 https://www.facebook.com/Learneating

2 https://www.facebook.com/foodnext.net

3 https://www.facebook.com/FoodQA/

4 https://www.facebook.com/wayneagrifoodlife/
